# Which is the best management for women with normal cervical cytologic findings despite positivity for non-16/18 high risk human papillomaviruses?

**DOI:** 10.3389/fpubh.2022.950610

**Published:** 2022-11-09

**Authors:** Ming Wu, Xiaotong Ma, Huiyang Li, Bijun Li, Chen Wang, Xiangqin Fan, Aiping Fan, Fengxia Xue

**Affiliations:** ^1^Department of Gynecology and Obstetrics, Tianjin Medical University General Hospital, Tianjin, China; ^2^Tianjin Key Laboratory of Female Reproductive Health and Eugenic, Department of Gynecology and Obstetrics, Tianjin Medical University General Hospital, Tianjin, China

**Keywords:** normal cytology, high-risk human papillomaviruses, risk discrimination, colposcopy, management

## Abstract

Women who test positive for the human papillomavirus (HPV) but have normal cytology constitute the predominant subgroup of patients in the screening population in the post-vaccination era. The distribution of HPV genotypes changed dramatically, which was attributable to an increase in HPV vaccination coverage. These changes have created uncertainty about how to properly manage women with normal cytology, non-HPV16/18 infections, or persistent infections. Current recommendations include retesting and continued surveillance in the absence of HPV16/18 infection. However, these are not always applicable. The ability to implement genotyping or incorporate HPV16/18 with some additional high-risk HPV (HR-HPV) types for triage and management with the aim of identifying type-specific risks in this population could be acceptable. When the next set of guidelines is updated, generating potential triage strategies for detecting high-grade cervical lesions, such as the p16/Ki67 cytology assay and other alternatives that incorporate genotyping with newer tests, should be considered. Current clinical management is shifting to risk-based strategies; however, no specific risk threshold has been established in this population. Importantly, innovative triage testing should be evaluated in combination with primary screening and management. Furthermore, there is an untapped opportunity to coordinate HPV genotyping in combination with colposcopic characteristics to modify risk in this group. Hence, providing a more personalized schedule through the efficient application of risk stratification and improving the detection of pre-cancer and cancer is an option worth exploring.

## Introduction

Human papillomavirus- (HPV-) based screening, either alone or in combination with cytology, is reported to be more sensitive than cytology alone in detecting high-grade squamous intraepithelial lesions (HSILs); this has reduced the number of subsequent screening rounds required and has been used in several settings (1–3). Because HPV testing has a lower specificity than cytology, the management protocol for women who are negative for intraepithelial lesion or malignancy (NILM) but positive for non-16/18 high-risk HPVs (HR-HPVs) remains a controversial issue in the current screening context. The majority of women with positive HPV test results are women who are NILM (4).

Current guidelines recommend that women who are NILM be referred for colposcopy if HPV16/18 is positive, and that those who are negative for HPV16/18 be retested within 1 year (5, 6). On the other hand, women who are NILM and positive for non-16/18 HR-HPVs continue to have an increased risk of HSIL (7). When a screening strategy based on a repeat test is applied to this group of women with poor attendance rates, loss to follow-up may be a relevant issue (8). However, this approach may not be generalizable across countries, particularly those with a high prevalence of non-16/18 HR-HPV genotypes (9). There is no established optimal screening management strategy in place for women who are NILM but positive for non-16/18 HR-HPVs.

Other triage options including incorporated cytology and HPV16/18/31/33/45 genotyping should be considered in countries with high rates of referral for colposcopy (8). The significance of HPV genotyping in the early detection of preneoplasia has been confirmed in some regions (10), and the introduction of the HR-HPV genotyping assay could improve the detection of early-stage HSIL, thus extending the time interval to retest women who are NILM beyond 1 year (10). Possible changes in the distribution of HPV genotypes have been observed in the post-vaccination era (11); likewise, the prevalence of HSIL associated with HPV16/18 has decreased over the years (12). Consequently, it is estimated that the national rollout of HPV vaccination will reduce the number of colposcopies by 10% within the first 3–4 years in developed countries (12). However, due to multiple barriers, HPV vaccines remain limited in low- and middle-income countries (particularly in Africa and Asia) (13). The development of novel methods of screening and triage, such as immunohistochemistry methylation biomarker tests and self-assessment kits, has addressed some of the needs in low-income settings (14).

In this review, we examined the need for colposcopy referral in women who are NILM but positive for non-16/18 HR-HPVs by evaluating the risk for precursor lesions relative to these types. We also summarized the current management strategies (and their challenges) for this population with the aim of providing a valuable reference for policymakers to tailor further alternative management strategies or strategies complementary to colposcopy.

## Challenges

### Overall prevalence

Women who are NILM but positive for HR-HPV account for most of the subjects with positive screening results encountered in routine practice, ranging from 6.7 to 14.9% (15–18). In the overall population, the distribution of HPV genotypes in a decreasing order of frequency is 16, 52, 31, 51, 18, 58, 39, 33, and 59 (19). HPV prevalence is determined by many factors, including race, age, hygiene habits, vaccination coverage, economic conditions, and availability and quality of health infrastructures in local settings. For example, the five most common HPV genotypes in developed and developing countries are 16, 53, 52, 51, and 31 and 16, 52, 58, 18, and 53, respectively (19). Furthermore, the distribution of HR-HPV genotypes varies between US (20) and European (21) populations. Other continents, such as Asia and Africa, have a high prevalence of non-16/18 HPVs, with slightly different orders of frequency for similar genotypes. The five most common HPV genotypes in Asian and African women who are NILM are 16, 52, 58, 18, and 33 and 16, 58, 52, 35, and 18, respectively (22, 23). The results are presented in Table 1. A systematic review of 42 African studies testing for HPV infection in 17,273 women who were NILM (mostly from eastern Africa, with some from western, southern, and northern Africa) found that the most common HPV genotypes were HPV16, 52, 35, 18, 58, 51, 45, 31, 53, and 56 in descending order (24). However, there were wide region-based variations in HPV infection rates. The highest prevalence was found in southern Africa, followed by eastern, western, and northern Africa (24). Other marked disparities in the distribution of HPV infection have been observed in different communities. In the Korean population, HPV16 was the most prevalent genotype, followed by HPV53, 70, 58, and 52 (25); in China, the descending order of HPV genotype frequency was 16, 58, 52, 53, 33, and 18 (26).

**Table 1:** Overall prevalence of most oncogenic types.

**HR-HPV genotypes**	**World**		**Africa**		**America**		**Asia**		**Europe**	
	**No. tested**	**HPV Prev% (95% CI)**	**No. tested**	**HPV Prev% (95% CI)**	**No. tested**	**HPV Prev% (95% CI)**	**No. tested**	**HPV Prev% (95% CI)**	**No. tested**	**HPV Prev% (95% CI)**
16	453,184	2.8 (2.8–2.9)	19,726	2.4 (2.2–2.6)	105,042	3.3 (3.2–3.4)	142,676	2.3 (2.2–2.4)	180,090	2.8 (2.7–2.8)
18	440,810	1.1 (1.1–1.1)	19,726	1.4 (1.2–1.5)	104,589	1.2 (1.1–1.3)	134,981	1.1 (1.0–1.1)	178,318	1.0 (0.9–1.0)
31	415,367	1.2 (1.2–1.2)	19,420	1.1 (0.9–1.2)	101,222	1.3 (1.2–1.3)	124,291	0.6 (0.6–0.6)	164,983	1.5 (1.4–1.6)
33	413,075	0.7 (0.7–0.7)	19,420	0.9 (0.8–1.0)	101,346	0.6 (0.6–0.7)	124,689	0.7 (0.7–0.8)	164,623	0.7 (0.7–0.7)
35	396,307	0.5 (0.5–0.5)	19,324	1.7 (1.5–1.9)	100,087	0.6 (0.5–0.6)	120,080	0.3 (0.3–0.4)	153,819	0.4 (0.3–0.4)
39	389,537	0.9 (0.8–0.9)	18,288	0.7 (0.6–0.8)	99,690	1.1 (1.0–1.2)	114,047	0.6 (0.6–0.7)	152,061	0.9 (0.8–0.9)
45	394,993	0.7 (0.7–0.7)	19,324	1.3 (1.2–1.5)	100,495	0.8 (0.8–0.9)	116,818	0.4 (0.4–0.4)	155,359	0.7 (0.7–0.8)
51	387,242	1.1 (1.1–1.1)	18,288	1.1 (1.0–1.3)	96,789	1.3 (1.2–1.4)	114,924	0.6 (0.6–0.7)	154,244	1.3 (1.2–1.4)
52	394,732	1.5 (1.4–1.5)	18,384	1.7 (1.6–1.9)	99,941	1.2 (1.2–1.3)	119,710	2.0 (1.9–2.0)	153,700	1.2 (1.1–1.2)
56	393,968	0.8 (0.7–0.8)	18,288	0.9 (0.7–1.0)	99,878	0.8 (0.8–0.9)	119,318	0.6 (0.6–0.7)	153,487	0.8 (0.8–0.9)
58	403,023	1.0 (1.0–1.0)	18,384	1.7 (1.5–1.9)	99,215	1.0 (1.0–1.1)	127,203	1.3 (1.2–1.3)	155,224	0.7 (0.6–0.7)
59	380,168	0.7 (0.7–0.7)	18,288	0.8 (0.6–0.9)	100,053	1.1 (1.0–1.1)	108,317	0.3 (0.3–0.4)	150,513	0.7 (0.6–0.7)

HR-HPV, high-risk human papillomavirus; No, number;Prev, prevalence.

Adjust from HPV and Related Diseases Report (19–23).

Several longitudinal and cross-sectional studies have reported a relatively high prevalence of HSIL in women who were NILM but positive for HR-HPV. The proportion of HSIL or more advanced lesions ranges from 5.40 to 20.39% (16, 27–29). Moreover, HSIL was detected in 7.5% (20/265) of women who were NILM but positive for HR-HPV during the follow-up period (9).

### Risk for progression to cervical preneoplastic lesions

Human papillomavirus is a major carcinogen in the uterine cervix. Although most HPV infections tend to regress, there is a risk of preneoplasia and cancer, particularly in women who are NILM but have non-16/18 HR-HPV infections. The fact that HPV16/18 poses a considerable risk for developing HSIL in women who are NILM is well-established in the literature. Women with HPV16/18 infections have a higher risk for HSIL and more advanced lesions than those with non-16/18 HPV infections (15, 30). Several observational studies have associated non-16/18 HR-HPVs with an increased risk to cervical preneoplasia (Table 2). A systematic review by Bonde et al. (31) summarized recent studies that used HPV genotyping to report the risk for HSIL. Two retrospective studies analyzed the odds ratios (ORs) for HSIL in women who were NILM but positive for HR-HPVs (26, 29). HPV genotyping is a strong predictor of oncogenic progression risk (30, 32, 33). Longitudinal studies have reported the progression rate of non-16/18 HR-HPVs (4, 18, 34). A recent meta-analysis synthesized the cumulative risk for cervical preneoplastic lesions by time for the most frequent non-16/18 HR-HPV genotypes (Table 3).

**Table 2:** Risk of progression to cervical preneoplastic lesions.

**HPV genotypes**	**Bonde et al. (31)**	**Wang et al. (29)**	**Zhang et al. (26)**
	**Risk of HSIL+ stratified by HPVs (≥30 y)**	**OR for HSIL+ (95% CI)**	**OR for HSIL+ (95% CI)**
16	~8 to 25%	3.576 (2.442–5.238)	16/18: 3.26 (2.41–4.40) 16/18/31/33: 4.21 (2.99–5.93) 16/18/31/33/52/58: 5.73 (3.30–9.97)
18	18,31 and 33: ~5 to 10%	1	
31		2.633 (1.205–5.753)	
33		2.386 (1.101–5.172)	
45	~1.7 to 3.7%	0.347 (0.045–2.670)	
52	~1.2 to 3.8%	1.819 (1.096–3.020)	
58	~0.4 to 1.4%	1.340 (0.749–2.395)	
35	~0.8 to 6.0%	1.461 (0.469–4.554)	NR
39	~0.8 to 3.1%	0.336 (0.100–1.130)	NR
51	~1.1 to 1.8%	0.463 (0.158–1.355)	NR
53	NR	0.090 (0.012–0.670)	NR
56	~0 to 0.8%	0.341 (0.101–1.149)	NR
59	~0 to 0.6%	0.217 (0.029–1.639)	NR
66	~0 to 1.2%	0.356 (0.082–1.542)	NR
68	~0 to 0.7%	0.302 (0.070–1.300)	NR

OR, odds ratio; HPV, human papillomavirus; HSIL, high-grade squamous intraepithelial lesions; NR, not reported.

**Table 3:** The progression rates by time for the most frequent non-16/18 HR-HPV genotypes.

**HPV type**	**Cumulative CIN2**+ **risk (%)**		
	**1 year**	**3 year**	**5 year**
	**(95% prediction interval)**	**(95% prediction interval)**	**(95% prediction interval)**
HR non−16/18	2.8 (0.0–10.2)	3.7 (0.0–11.0)	4.6 (0.0–11.8)
31	4.1 (0.0–11.4)	7.5 (0.0–14.6)	10.8 (3.2–17.8)
33	4.3 (0.0–11.6)	8.1 (0.3–15.2)	11.7 (4.0–18.8)
35	3.0 (0.0–10.4)	4.5 (0.0–11.7)	5.8 (0.0–13.1)
45	3.2 (0.0–10.5)	4.8 (0.0–12.1)	6.4 (0.0–13.6)
51	3.1 (0.0–10.4)	4.6 (0.0–11.8)	6.0 (0.0–13.2)
52	3.3 (0.0–10.6)	5.2 (0.0–12.4)	7.1 (0.0–14.2)
58	3.3 (0.0–10.6)	5.2 (0.0–12.5)	7.1 (0.0–14.3)
59	3.1 (0.0–10.4)	4.5 (0.0–11.8)	6.0 (0.0–13.2)

Adjust from Malagón et al. (32).

According to HPV genotypes, different age groups have different risks of progression over time (35). Moreover, the prevalence and risk for HSIL decline with increasing age in women who are NILM (1, 36). However, when an age cutoff of 40 years was used, the prevalence of HSIL in younger women who were NILM was significantly higher than that in older women, and the group of older women conferred a lower risk for cervical precursor lesions and a higher risk for cancer compared to the group of younger women (27).

### Risk for HPV persistence with normal cytology

The different oncogenic potentials of genotypes confer disparate risks for cervical precancerous lesions in women who are concurrently NILM and HPV positive. However, an HPV infection must be persistent to cause precursor lesions and cancer. The natural history of HPV infection is well-documented. It has been demonstrated that women who are NILM but have HPV persistence after a baseline HPV-positive test have an increased risk of HSIL. The rate of oncogenic development in natural course models of precancerous lesions and cancer is a crucial parameter because it determines the age at which models predict that HSIL will be identified during screening (32). Women who have HPV persistence after a baseline HPV-positive test and are NILM tend to develop cervical lesions at a shorter interval than that is recommended by guidelines for specific follow-up testing (37). Prospective, high-quality follow-up studies will be beneficial, particularly in determining the proper follow-up interval for this group.

Human papillomavirus genotypes vary substantially in terms of cumulative risk and annual rate of progression to pre-cancer (38). Women who are NILM but have persistence of the same HPV genotype have a higher risk of HSIL than those who have had a prior infection eliminated only to re-infect with a different HPV genotype (39). HPV persistence is strongly associated with the occurrence of cervical preneoplasia, but this association also increases in cases of infection with specific genotypes (37, 40). The most common genotypes in women who were NILM were HPV16, 52, 58, and 33, and the majority of subjects developed cervical preneoplasia within 24 months of HR HPV persistence (37).

High-risk HPV persistence is a crucial contributor to the pathophysiology of cervical lesions (precancerous) and cancer, with preneoplastic lesions taking several years to develop (41). However, most patients undergo clinical HPV clearance within a few months, indicating that other potential cofactors, such as host and vaginal microbiome disturbances, may be involved in the progression of cervical carcinogenesis. There is evidence that both host innate and adaptive immune responses, which are thought to be critical for viral clearance, can be inhibited by oncogenic HPV types, thus dampening the host immune response (42). Epidemiological links have identified relationships between vaginal dysbiosis and HPV-related diseases (particularly bacterial vaginosis), which are relevant to the risk factors involved in the initiation and development of HPV infection (43–46). The relationships among vaginal lactobacillus, HPV-related diseases, and HPV clearance have also been characterized. A trend toward slower disease regression was observed in women with non-lactobacillus predominance at baseline than in those with *Lactobacillus crispatus* or *Lactobacillus iners* predominance at the time of HSIL diagnosis (47).

Therefore, recognizing the natural history of precancerous lesions, cancer, and other potential cofactors is of great clinical importance for determining specific risk thresholds and proper clinical screening intervals, particularly in women who are NILM positive for non-16/18 HR-HPVs. In addition, it has been observed that the maintenance of the vaginal microbiome and the improvement of vaginal microecology favor the regression of cervical lesions through a prebiotic effect (48). Elucidating the potential for therapeutic manipulation of vaginal microbiota holds promise for improving outcomes regarding HPV clearance and cervical lesions.

As discussed earlier, these data highlight that women who are NILM but positive for non-16/18 HR-HPVs are still at risk of developing precancerous lesions and cancer beyond 3 or 5 years. The differences in the likelihood of developing precursor lesions and cancer depend primarily on the carcinogenic potential of different genotypes and on geographic and ethnic variations.

### Limitations on current management

The current major guidelines for the management of women who are NILM but positive for non-16/18 HR-HPVs are reported in Table 4. Current triage methods for these women include referral for colposcopy, retesting after 1 or more years, HPV genotyping, and other new approaches.

**Table 4:** Summary of the current major guidelines in the management of negative for intraepithelial lesion or malignancy (NILM) women positive for non-16/18 HR-HPVs.

**Body**	**Country**	**Year**	**Management**	
			**HPV16/18**	**HR non-16/18 HPVs**
ACS (3)	USA	2012	Refer to colposcopy	Repeat cotesting 12 months
ACOG (5)	USA	2016	Refer to colposcopy	Cotested in 12 months
ASCCP (6)	USA	2012	Refer to colposcopy	Repeat co-testing in 1 year
ASCCP (49)	USA	2020	Use risk discrimination to determine the best course of action for
			different screening results
NCSP (50)	Australia	2020	Refer to colposcopy	Cotested in 12 months
JSOG and JAOG (51)	Japan	2019	Refer to colposcopy	-
ESGO-EFC (52)	Europe	2020	Retested for hpv in 12 months or for cytology in 6–12 months
NHS (53)	England	2020	Have the hpv test repeated at 12 months
DHC (54)	Netherlands	2019	Follow-up testing of HPV-positive women with normal cytology

ACOG, American College of Obstetricians and Gynecologists; ACS, American Cancer Society; ASCCP, American Society for Colposcopy and Cervical Pathology; DHC, Dutch Health Council; ESGO-EFC, European Society of Gynecologic Oncology and the European Federation of Colposcopy; NHS, National Health Service; JSOG, Japan Society of Obstetrics and Gynecology; JAOG, Japan Association of Obstetricians and Gynecologists; NCSP, Australian National Cervical Screening Program.

Partial HPV triage of normal cytologic findings offers an effective approach to discriminate between women with HPV16/18 infections and those with non-16/18 infections; however, the implementation of this strategy is limited by the higher distribution of non-16/18 HR-HPVs in women who are NILM. HPV16/18 genotyping is unlikely to be effective in triaging women who are NILM but positive for HR-HPV; it has a positive predictive value (PPV) of 11% and is not generalizable to younger women who have been vaccinated for HPV. Conversely, the HPV E6/E7 messenger ribonucleic acid (mRNA) assay may play a role in risk management in this population (55). The data reported by Bonde et al. (31) supported the concept that the management of women positive for HPV18, 31, 33, 45, 52, or 58 could be developed according to regional risk thresholds, including retesting and colposcopy, based on a genotype risk continuum. Risk stratification based on HPV genotyping may not be generalizable to other regions in population-based screening. However, infection is cleared within 3 years in approximately three-fourths of women who are NILM but positive for non-16/18 HR-HPVs, indicating that the current management strategy of annual repeat testing and referral after 2 years may be inaccurate (56).

Another unresolved problem is the management of women who are NILM but have HPV persistence after two rounds of triage (4), including the triage of older women with HPV persistence. Recognizing that women who are NILM but have non-16/18 HR-HPV persistence are still at risk for precancerous lesions; however, the utility of the current strategy is confined to a higher risk in women who have type-specific persistence of non-16/18 genotypes. This is especially true for younger NILM women, who had a significantly higher HSIL prevalence than older women (27). When no precursor lesion is detected at the initial screening, there is no agreement on the duration of follow-up on non-HPV16/18 persistence before referral to colposcopy or treatment. Moreover, as the frequency of HSIL in older women is very low and most diseases are related to other genotypes, should these women be offered excision to remove the disease? Should they be screened yearly until they become HR-HPV negative or can the decision to cease screening be based on the specificity of persistent normal cytology? These questions remain unanswered (1). When modern assays are utilized before guideline panels can deliver an alternative to colposcopy for these women, additional information on the long-standing risk for HSIL according to HPV type-specific persistence of genotypes other than 16/18 in women who are NILM is needed.

### Colposcopy

The role of colposcopy in predicting cervical disease and in cervical cancer screening remains controversial. Colposcopic impression of underlying disease appears to be less effective in the presence of non-16/18 HPVs and in those over the age of 50 (1). Since the prevalence of HPV16/18 is decreasing, the risk for progression in women infected with non-16/18 HR-HPVs is higher in HPV16/18-vaccinated populations. Accordingly, the performance of colposcopy in predicting precancerous lesions may be poor. Aydin et al. (28) suggested that, among cytology-negative women, a lower PPV for HSIL was observed in subjects positive for HPVs other than 16/18 than in women positive for HPV16/18 (5.1%; 95% confidence interval (CI), 1.9–12.1 vs. 14.7; 95% CI, 8.4–24.3). Furthermore, cytologic findings, genotypes, age, and operator experience all play an important role in the overall diagnostic performance of colposcopy (57).

## Opportunities

### New options for colposcopy

According to the literature, vaccination will not fully avert an increase in colposcopy referrals and the detection of preneoplasia in the first 2–3 years of HPV-based screening. Given the herd effect of the HPV vaccine, the amount is thought to be reduced after 5–6 years, and then by approximately 20% (12, 58). It is also predicted that the increase in HR types 31/33/45/52/58 needs to be re-evaluated once the nanovalent vaccine effect becomes more reliable (12). Consequently, because the effect of vaccination on colposcopies and cervical preneoplasia will be gradual, ways of delivering a more personalized schedule by applying risk stratification and thus improving the identification of precancerous lesions and cancer are worth exploring.

In screening management settings, women who are NILM may have a greater association with smaller and undetectable lesions than those who have cytological abnormalities (59). HPV16-associated premalignancy appears to progress faster (60) than non-16 HPV-associated premalignancy (17, 61, 62). Given the differences in non-16/18 HPV genotypes, their correlation with HSIL, and their colposcopic performance, it is still possible to coordinate HPV genotyping in combination with colposcopy to refine risk in the NILM group positive for non-16/18 HR-HPVs in the foreseeable future, although this strategy is underutilized.

### Cytologically false-negative results

Morphological appearance is less sensitive and highly subjective and can therefore lead to false-negative results (55, 63). Women infected with non-16/18 HR-HPVs or mixed-HPV genotypes are more frequently associated with misleading lower-grade cytomorphology than those infected with single HPV16/18 genotypes, accounting for false-negative results or underdiagnosis on Pap tests (64).

The interpretation of cytomorphology is influenced by sampling, different cytologic presentations, and other factors (e.g., age and physical or pathological changes). The paucity of abnormal cells is the major cause of cytological differences, and variations in interpretation are affected by opaque inflammation (55, 65). Furthermore, other confounders (i.e., low cellularity, obscured nuclear features due to nuclear crowding and overlapping, presence of pale immature metaplastic cells, and a lack of diagnostic cells) contribute to low sensitivity of cytology (55).

The limitations of cytological interpretation mentioned earlier highlight the need to add an objective screening method with higher sensitivity, such as the HPV assay and p16/ Ki-67 dual-stained (DS) cytology. Combining cytology and HPV testing is intended to improve the detection of cervical abnormalities in the future (64). The addition of DS also ensures good clinical performance in detecting cervical precursor lesions and cancer, with sensitivity and specificity similar to HR-HPV and cytology (66). Over 5 years, DS screening provides better long-term risk discrimination than cytology alone (67). A prospective observational trial indicated that DS cytology was considerably more sensitive and specific than HPV16/18 genotyping combined with cytological triage of non-16/18 HPV genotypes in predicting the risk for HSIL (68). Clarke et al. (67) demonstrated that HPV-positive women with DS-negative findings conferred a significantly lower 5-year risk of HSIL than women who were NILM (8.5%; 95% CI, 6.5–11.1% vs. 12.3%; 95% CI, 9.8–15.4%; *p* = 0.04). The low risk for cervical preneoplasia in DS-negative women allows safe extension of follow-up intervals to 3 years. Moreover, Li et al. (69) compared the Dalton (DS kit produced by Hangzhou Dalton Biosciences) and CINtec^®^ PLUS p16/Ki-67 dual stain in HPV-positive Chinese women and found that DS cytology was valuable for triaging HPV-positive women.

### Triage and management

Cervical lesions linked to non-16/18 HR-HPV infections represent the predominant subgroup in the screening population due to the dramatic change in the distribution of HR-HPV genotypes among vaccinated women (32, 70–72). Developing a highly effective test for detecting precursor lesions and cancers, as well as providing individually specific management approaches, which will improve the survival of women, are of paramount relevance in population screening. The updated 2019 American Society for Colposcopy and Cervical Pathology guidelines reflect a significant shift toward risk-based screening to identify women at risk for precancerous lesions. This risk-based management approach delivers a more personalized schedule for population-based screening of women. Table 5 shows the distribution of HPV genotypes with specific applications to non-16/18 HPV genotypes in different geographical areas.

**Table 5:** Specific application to non-16/18 HPV genotypes in different geographical areas.

**Study**	**Country**	**Specific application**	**Population**	**HPV testing assay**	**Distribution of HPV genotypes in descending order**	**Main findings**	**Specific application to non-16/18 HPV genotypes in NILM women**
Khuna-mornpong et al. (78)	Thailand	16/18+addition	*N* = 226, age ≥ 25 y, NILM women positive for HPV	HC2 and Linear Array (Roche Molecular System, Inc., Branchburg, NJ, USA)	HPV52 (74, 32.7%), HPV39 (38, 16.8%), HPV68 (25, 11.1%), HPV16 (24, 10.6%), HPV51 (17, 7.5%), HPV56 (17, 7.5%), HPV31 (15, 6.6%), HPV18 (12, 5.3%)	The rate of histologic HSIL+ in each genotype was above 10% for HPV16 (16.7%), HPV31 (13.3%), HPV52 (14.9%), and HPV58 (16.7%)	Addition of genotyping for HPV52/58 or HPV31/52/58 to HPV16/18 in NILM women
Stoler et al. (76, 77)	USA	16/18+addition	*N* = 22,383, age ≥ 30 y, NILM women	Onclarity (Becton, Dickinson and Company, BD Life Sciences-Diagnostic Systems)	HPV16/18: 423 (1.9%) HPV18/45: 208 (0.9%) HPV16/18/45: 538 (2.4%)	HPV 16, 18, 45, and the other 11 genotypes had CIN3 or higher risks of 6.9, 2.6, 1.1, and 2.2%, respectively	Detection of individual HPV 16, 18, or 45
Zhang et al. (26)	China	16/18+addition	*N* = 2,665, age ≥ 25 and ≤ 65 y, NILM women positive for HPV	HPV GenoArray test kit (Hybribio Ltd., Hong Kong).	HPV16 (874, 30.2%), HPV58 (452, 15.6%), HPV52 (395, 13.6%), HPV53 (380, 13.1%), HPV18 (224, 7.7%), HPV33 (208, 7.2%)	OR for HSIL+ (95% CI): HPV16/18: 3.26 (2.41–4.40) HPV16/18/31/33: 4.21 (2.99–5.93) HPV16/18/31/33/52/58: 5.73 (3.30–9.97)	Addition of genotyping for HPV31/33 to HPV16/18
Xu et al. (30)	China	Domestic HPV Testing	*N* = 2,180, age 30–64 years, NILM women	DH3 (a novel domestic high-risk HPV testing based on hybrid capture)	HPV other HPV+: 140 HPV16/18+: 30 HPV16/18& other HPV+: 15	The cumulative absolute risk for the development of CIN2+ was 37.8% for HPV 16/18 positive women, followed by HR-HPV positive (14.6%), other HR-HPV positive (11.0%) and HPV negative (0.3%) in 3 years	DH3 HPV assay demonstrated excellent clinical performance against CIN2+ detection and utility of risk stratification
Song et al. (73)	China	extended genotyping +p16^INK^ immune-staining	*N* = 2,731, age 30–64 years, NILM women positive for HPV	Seq (BGI Shenzhen, Shenzhen, China)	HPV16 (23.7%), HPV52 (16.7%), HPV58 (14.0%), HPV51 (11.1%), HPV18 (10.5%)	The p16 positivity rates among three genotype strata were 40.2% (HPV16/33), 22.9% (HPV58/31/35), and 18.8% (other 9 types), respectively, increasing with the elevation of hierarchical types (*p* < 0.0001); three triage strategies were favorable in sensitivity and/or specificity to the ‘HPV16/33+' strategy: p16+; ‘HPV16+ or HPV33/58/31/35 + &p16+'; HPV16/18/31/33/45/52/58 + &p16 +	Genotyping for HPV16/33 could be utilized; p16 immunostaining, either alone or combined with extended genotypes, is more effective than HPV genotypes alone
Wright et al. (68)	USA	DS	*N* = 4,927 HPV-positive women NILM women: 3,090	Cobas 4,800 and cobas 6,800/8,800 HPV (Roche Molecular Systems, Inc, Pleasanton, CA)	in NILM women: 12 other HPV+ and DS+: 750 (34.2%) 12 other HPV+ and DS-: 1,444 (65.8%)	In 12 “other” HPV-positive women, sensitivity of DS for ≥CIN2 and ≥CIN3 at baseline was 83.0 and 86.0%, respectively, significantly higher as compared to the respective sensitivity estimates of cytology	DS is effective for triage of HPV-positive women, either alone or when combined with partial HPV genotyping

HPV, human papillomavirus; NILM, negative for intraepithelial lesions or malignancies; CIN, cervical intraepithelial neoplasia; DS, p16/Ki-67 dual-stained cytology.

As HPV testing assays become more well-established, the genotype and duration of infection can allow for more accurate risk evaluations (38). HPV genotyping, which is considered a type of triage and includes partial and extended genotyping as well as combining the most oncogenic with additional genotypes, can be used to optimize the management of women who are NILM (73, 74).

The data presented by Arbyn et al. (71) confirm that implementing partial genotyping schedules allows for a better balance of the benefits of screening compared with colposcopy referral because women with “other HR-HPV type” infections can be managed differently from those with HPV16/18 positivity. Management stratified by the presence of HPV16/18 could be more efficient (at the expense of small losses) in the detection of cervical preneoplasia. In contrast, data from the HPV Pilot Steering Group in England provided very little additional information on the benefits of HPV16/18 genotyping (75). Moreover, the strategy of recalling women who are NILM and have HPV persistence after 2–3 years and then referring those who develop moderate+ cytology and type-specific persistence for colposcopy could be optional (56).

Extended genotyping is an important part of precise risk evaluation and appropriate clinical management and offers the opportunity to apply risk discrimination and tailor management (e.g., 12-vs. 24-month follow-up or retest). Increasing evidence supports the concept that extended HPV genotyping is effective for risk stratification in women who are NILM (70). The study by Mark et al. (76) suggested that risk-informed management was supported by accurate risk strata through extended genotyping. Their results showed that HPV16 and 31 exceeded the risk threshold for colposcopy referral, HPV18, 33/58, and 52 formed an intermediate-risk band, and HPV45, 51, 35/39/68, and 56/59/66 formed the lowest-risk band. The management of women who are positive for intermediate- or lower-risk genotypes may evolve based on refined risk evaluations and clinical factors. Given the recent US Food and Drug Administration (FDA) approval for extended genotyping, guidelines regarding HPV typing are required (74). Further colposcopy referral based on extended genotyping may be explicitly exploited to rapidly change the prevalence of HPV genotypes in young, vaccinated women.

Multiple studies have illustrated different approaches to triage for women who are NILM but positive for non-16/18 HR-HPVs, as presented in Table 5. A longitudinal study of 33,858 US women undergoing routine screening suggested that the efficacy of overall HPV testing and testing for specific genotypes (16, 18, and 45) was clinically validated for co-testing in women who were NILM (77). In a study carried out in northeastern China, Zhang et al. (26) recommended that HPV31/33 genotyping be added to HPV16/18 genotyping in triaging the NILM/HPV+ group; this recommendation supports immediate colposcopy for women with HPV16/18/31/33 infections. Moreover, a cross-sectional study of 5,456 women in Thailand found that adding HPV52/58 genotyping to HPV16/18 genotyping might effectively improve the performance of triage in detecting histological HSIL (78). Therefore, the ability to incorporate the most carcinogenic types into additional genotypes may be acceptable for the triage and management of women who are NILM to identify type-specific risks.

In addition, the DS cytology assay, which has been approved as a triage test by the FDA, is more accurate in detecting preneoplasia in HPV-positive women than cytology alone (74). This combined assay has recently been approved as an HPV screening test because of its minimal invasiveness, greater accuracy in detecting precancerous lesions, and the elimination of unnecessary colposcopy referral (73, 74, 79–81). DS, either alone or in combination with HPV16/18 or extended genotyping, is the preferred technique for valuable triage of colposcopy in HPV-positive women (82, 83). Furthermore, DS is a desirable triage option for vaccinated women with an increased frequency of HPV16/18 infections because it performs well in HPV16/18-negative women in terms of risk stratification (80). Moreover, Song et al. (73) reported that p16 immunostaining detected 83.1% (79.2%) of underlying CIN2+ (CIN3+) in 2,731 women who were NILM but positive for HPV, when extended genotypes were utilized. These data suggest that p16 staining is more beneficial than HPV genotyping alone in triaging this population (73).

Several innovative screening assays have been introduced, including HPV triage protocols (HPV mRNA and DH3), surrogates for cytology (automated DS cytology), biomarkers (viral load and deoxyribonucleic acid (DNA) methylation), and other technologies (deep learning-assisted visual triage and self-sampling technology). For instance, in China, the DH3 HPV assay, a newly developed RNA–DNA hybrid capture-based technique similar to the Hybrid Capture 2 (HC2), has been utilized (in combination with HPV16/18 genotyping) for cervical cancer screening (30, 84, 85). The clinical performance of the assay in identifying HSIL was comparable to that of the Cobas 4800 HPV and HC2 tests (84, 85). Comparisons between DH3 and Cobas 4800 revealed similar results in terms of HSIL detection (93.3 vs. 91.1%, 91.2 vs. 91.8%, 12.0 vs. 12.5%, and 99.9 vs. 99.9%, for sensitivity, specificity, PPV, and negative predictive value, respectively) (85). Similarly, the observed agreement between the assay and HC2 was 99.2% (κ = 0.938); the reported sensitivity of DH3 for HSIL was equal to that of HC2 (98.67%) (84). Moreover, this assay showed superior clinical performance in terms of HSIL detection and risk discrimination in Chinese women who were NILM (30). HPV16/18 genotyping showed high sensitivity (91.7%) and specificity (85.7%) for HSIL detection (30). Due to its concurrent HPV16/18 genotyping and non-polymerase chain reaction- (PCR-) based benefits, the DH3 HPV assay may have triage applications in developing countries. The FAM19A4/miR124-2 methylation test, another novel technology, was developed to screen and diagnose (a triage test). It is thought to have the potential to supplement cytology as a triage modality in HPV-based screening contexts (86–88). A Dutch study evaluating the cross-sectional performance of this test showed similar sensitivity and specificity between the test and cytology (71.3 vs. 76.0% and 78.3 vs. 87.0%, respectively) (87). This methylation test is highly sensitive for HSIL compared with the combination of cytology and HPV genotyping (93 vs. 86%) (89). There is evidence that a negative FAM19A4/miR124-2 methylation test has similar or better long-term performance than normal cytologic findings for detecting cervical precancerous lesions and cancer (86, 87). In addition, methylation and/or HPV genotyping in HPV-positive women with low-grade cytology can reduce the number of colposcopy referrals (90). Further evidence is needed for the cross-sectional and long-term evaluation of the test in women who are NILM but positive for non-16/18 HR-HPVs. Moreover, it has been demonstrated that the HR-HPV mRNA APTIMA assay, which detects 14 HR-HPV types, may be a beneficial triage modality in HPV DNA-positive postmenopausal women with negative cytological findings (65).

The anticipation of these screening options will provide a promising insight into developing a proper screening program in women who are NILM in collaboration with cytology. Nevertheless, clinical evidence for these performances is still being studied, particularly in association with risk prediction during a follow-up.

## Conclusion

In conclusion, our review supports the combination of various HPV genotyping assays and recommends that HPV persistence and regional contexts be considered in overall assessment, follow-up, and management in women who are NILM, which will allow for the evaluation of genotype-specific test outcomes and will support risk discrimination or tiered risk stratification. It will also reduce the burden of colposcopy referral as clinicians will be able to better distinguish women with HPV persistence from those with incidentally detected HPV infection. It is noteworthy to consider extended genotyping based on different natural courses and risk profiling (38). Moreover, considering the regional disparities and distinguishable oncogenicity of HPV types, we suggest developing a screening algorithm based on risk-informed HPV genotyping that combines clinical factors and colposcopy impression. When used sequentially and in coordination, this will classify women based on risk (from the highest to lowest likelihood of developing precancerous lesions), guide colposcopy referral, and limit overtreatment in the population. Furthermore, it is possible that the introduction of new, accurate, and precise biomarkers will replace traditional cytology in the detection of precancerous conditions. Perhaps emerging innovative advancements will allow risk evaluation through screening or triage tests, rather than solely through colposcopy-guided biopsy.

## Author contributions

MW conducted the literature search and drafted the first manuscript. XM, HL, BL, CW, XF, and AF contributed significantly to writing and critically revising the review. FX conceived and designed the structure, framework of this review, and critically revised the work. All authors were involved in the study design and read and approved the final manuscript.

## Funding

This work was funded by the Tianjin Municipal Science and Technology Commission Special Foundation for Science and Technology Major Projects in Control and Prevention of Major Diseases (Grant No. 18ZXDBSY00200).

## Conflict of interest

The authors declare that the research was conducted in the absence of any commercial or financial relationships that could be construed as a potential conflict of interest.

## Publisher's note

All claims expressed in this article are solely those of the authors and do not necessarily represent those of their affiliated organizations, or those of the publisher, the editors and the reviewers. Any product that may be evaluated in this article, or claim that may be made by its manufacturer, is not guaranteed or endorsed by the publisher.

## References

[1] Tidy JA Lyon R Ellis K Macdonald M Palmer JE. The impact of age and high-risk human papillomavirus (hrHPV) status on the prevalence of high-grade cervical intraepithelial neoplasia (CIN2+) in women with persistent hrHPV-positive, cytology-negative screening samples: a prospective cohort study. BJOG. (2020) 127:1260–7. 10.1111/1471-0528.16250 32279427

[2] Thomsen LT Kjaer SK Munk C Ornskov D Waldstrom M. Benefits and potential harms of human papillomavirus (HPV)-based cervical cancer screening: a real-world comparison of HPV testing versus cytology. Acta Obstet Gynecol Scand. (2021) 100:394–402. 10.1111/aogs.14121 33566361

[3] Saslow D Solomon D Lawson HW Killackey M Kulasingam SL Cain JM. American cancer society, American society for colposcopy and cervical pathology, and American society for clinical pathology screening guidelines for the prevention and early detection of cervical cancer. J Low Genit Tract Dis. (2012) 16:175–204. 10.1097/LGT.0b013e31824ca9d5 22418039 PMC3915715

[4] Gilham C Sargent A Peto J. Triaging women with human papillomavirus infection and normal cytology or low-grade dyskaryosis: evidence from 10-year follow up of the ARTISTIC trial cohort. BJOG. (2020) 127:58–68. 10.1111/1471-0528.15957 31541495 PMC6916371

[5] COP BG. Practice Bulletin No. 168: cervical cancer screening and prevention. Obstet Gynecol. (2016) 128:e111–30. 10.1097/aog.0000000000001708 27661651

[6] Massad LS. EM, Huh WK, Katki HA, Kinney WK, Schiffman M, et al. Updated consensus guidelines for the management of abnormal cervical cancer screening tests and cancer precursors. J Low Genit Tract Dis. (2012) 2013:S1–27. 10.1097/LGT.0b013e318287d329 23519301

[7] Polman NJ Veldhuijzen NJ Heideman DAM Snijders PJF Meijer CJLM Berkhof J. HPV-positive women with normal cytology remain at increased risk of CIN3 after a negative repeat HPV test. Brit J Cancer. (2017) 117:1557–61. 10.1038/bjc.2017.309 28881359 PMC5680458

[8] Rijkaart DC Berkhof J van Kemenade FJ Coupe VM Hesselink AT Rozendaal L. Evaluation of 14 triage strategies for HPV DNA-positive women in population-based cervical screening. Int J Cancer. (2012) 130:602–10. 10.1002/ijc.26056 21400507

[9] Song JS Kim EJ Choi J Gong G Sung CO. Significance of HPV-58 infection in women who are HPV-positive, cytology-negative and living in a country with a high prevalence of HPV-58 infection. PLoS One. (2013) 8:e58678. 10.1371/journal.pone.0058678 23505548 PMC3591398

[10] Dong BH Zou HC Mao XD Su YY Gao HJ Xie F. Effect of introducing human papillomavirus genotyping into real-world screening on cervical cancer screening in China: a retrospective population-based cohort study. Ther Adv Med Oncol. (2021) 13:939. 10.1177/17588359211010939 33995595 PMC8107662

[11] Freire-Salinas J Benito R Azueta A Gil J Mendoza C Nicolás M. Genotype distribution change after human papillomavirus vaccination in two autonomous communities in Spain. Front Cell Infect Microbiol. (2021) 11:633162. 10.3389/fcimb.2021.633162 34631594 PMC8493034

[12] Giannella L Delli Carpini G Di Giuseppe J Bogani G Gardella B Monti E. Trend of HPV 16/18 genotypes in cervical intraepithelial neoplasia grade 3: data for 2007–2018. Infect Drug Resist. (2021) 14:3763–71. 10.2147/IDR.S326851 34557001 PMC8453441

[13] Baussano I Sayinzoga F Tshomo U Tenet V Vorsters A Heideman DAM. Impact of human papillomavirus vaccination, Rwanda and Bhutan. Emerg Infect Dis. (2021) 27:1–9. 10.3201/eid2701.191364 33350922 PMC7774553

[14] Bogdanova A Andrawos C Constantinou C. Cervical cancer, geographical inequalities, prevention and barriers in resource depleted countries. Oncol Lett. (2022) 23:113. 10.3892/ol.2022.13233 35251344 PMC8850967

[15] Wright TC Jr Stoler MH Sharma A Zhang G Behrens C Wright TL. Evaluation of HPV-16 and HPV-18 genotyping for the triage of women with high-risk HPV+ cytology-negative results. Am J Clin Pathol. (2011) 136:578–86. 10.1309/AJCPTUS5EXAS6DKZ 21917680

[16] Tao X Zhang H Li J Zhang H Xiao J Zhang L. Prevalence of HPV-16/18 genotypes and immediate histopathologic correlation results in a Chinese population with negative cytology and positive high-risk HPV testing. Cancer Cytopathol. (2019) 127:650–7. 10.1002/cncy.22180 31532582

[17] Safaeian M Schiffman M Gage J Solomon D Wheeler CM Castle PE. Detection of precancerous cervical lesions is differential by human papillomavirus type. Cancer Res. (2009) 69:3262–6. 10.1158/0008-5472.CAN-08-4192 19351830 PMC3155840

[18] Song F Du H Xiao A Wang C Huang X Liu Z. Type-specific Distribution of Cervical hrHPV infection and the association with cytological and histological results in a large population-based cervical cancer screening program: baseline and 3-year longitudinal data. J Cancer. (2020) 11:6157–67. 10.7150/jca.48357 32922555 PMC7477419

[19] Bruni L AG Serrano B Mena M Collado JJ Gómez D Muñoz J Bosch FX de Sanjosé S. ICO/IARC Information Centre on HPV and Cancer (HPV Information Centre). Human Papillomavirus and Related Diseases Report in the World. Summary Report 22 October 2021. Available online at: www.hpvcentrenet (accessed 22 October 2021).30699273

[20] Bruni L AG Serrano B Mena M Collado JJ Gómez D Muñoz J Bosch FX de Sanjosé S. ICO/IARC Information Centre on HPV and Cancer (HPV Information Centre). Human Papillomavirus and Related Diseases in Americas. Summary Report 22 October 2021. Available online at: www.hpvcentrenet (accessed 22 October 2021).30699273

[21] Bruni L AG Serrano B Mena M Collado JJ Gómez D Muñoz J Bosch FX de Sanjosé S. ICO/IARC Information Centre on HPV and Cancer (HPV Information Centre). Human Papillomavirus and Related Diseases in Europe. Summary Report 22 October 2021. Available online at: www.hpvcentrenet (accessed 22 October 2021).30699273

[22] Bruni L AG Serrano B Mena M Collado JJ Gómez D Muñoz J Bosch FX de Sanjosé S. ICO/IARC Information Centre on HPV and Cancer (HPV Information Centre). Human Papillomavirus and Related Diseases Report in Asia. Summary Report 22 October 2021. Available online at: www.hpvcentrenet (accessed 22 October 2021).30699273

[23] Bruni L AG Serrano B Mena M Collado JJ Gómez D Muñoz J Bosch FX de Sanjosé S. ICO/IARC Information Centre on HPV and Cancer (HPV Information Centre). Human Papillomavirus and Related Diseases in Africa. Summary Report 22 October 2021. Available online at: www.hpvcentrenet (accessed 22 October 2021).30699273

[24] Ogembo RK Gona PN Seymour AJ Park HS Bain PA Maranda L. Prevalence of human papillomavirus genotypes among African women with normal cervical cytology and neoplasia: a systematic review and meta-analysis. PLoS ONE. (2015) 10:e0122488. 10.1371/journal.pone.0122488 25875167 PMC4396854

[25] Seong J Ryou S Lee J Yoo M Hur S Choi BS. Enhanced disease progression due to persistent HPV-16/58 infections in Korean women: a systematic review and the Korea HPV cohort study. Virol J. (2021) 18:188. 10.1186/s12985-021-01657-2 34535177 PMC8447749

[26] Zhang J Zhang D Yang Z Wang X Wang D. The role of human papillomavirus genotyping for detecting high-grade intraepithelial neoplasia or cancer in HPV-positive women with normal cytology: a study from a hospital in northeastern China. BMC Cancer. (2020) 20:443. 10.1186/s12885-020-06935-w 32429919 PMC7236298

[27] Liu Q Zhou X Zhang X Strickland AL Zheng W Chen H. HPV Genotype specific and age stratified immediate prevalence of cervical precancers and cancers in women with NILM/hrHPV+: a single center retrospective study of 26,228 cases. Cancer Manag Res. (2021) 13:6869–77. 10.2147/CMAR.S328279 34512026 PMC8421554

[28] Aydin S Oncu HN Arici DS. Diagnostic performance of immediate colposcopy among women with high-risk human papillomavirus (HPV) other than HPV 16/18 and normal cytology. J Obstet Gynaecol Res. (2021) 47:720–5. 10.1111/jog.14597 33314453

[29] Wang Z Liu T Wang Y Gu Y Wang H Liu J. Risk of cervical lesions in high-risk HPV positive women with normal cytology: a retrospective single-center study in China. Infect Agent Cancer. (2020) 15:34. 10.1186/s13027-020-00291-x 32477424 PMC7240930

[30] Xu HF Liu Y Luo YL Zhao DM Jia MM Chen PP. The risk stratification for cervical cancer and precursors of domestic HPV testing with HPV 16/18 genotyping in women with NILM cytology in Central China: a cohort study. Front Oncol. (2021) 11:716762. 10.3389/fonc.2021.716762 34671550 PMC8521162

[31] Bonde JH Sandri MT Gary DS Andrews JC. Clinical utility of human papillomavirus genotyping in cervical cancer screening: a systematic review. J Low Genit Tract Dis. (2020) 24:1–13. 10.1097/LGT.0000000000000494 31714325 PMC6924950

[32] Malagon T Volesky KD Bouten S Laprise C El-Zein M Franco EL. Cumulative risk of cervical intraepithelial neoplasia for women with normal cytology but positive for human papillomavirus: systematic review and meta-analysis. Int J Cancer. (2020) 147:2695–707. 10.1002/ijc.33035 32363604

[33] Del Mistro A Adcock R Carozzi F Gillio-Tos A De Marco L Girlando S. Human papilloma virus genotyping for the cross-sectional and longitudinal probability of developing cervical intraepithelial neoplasia grade 2 or more. Int J Cancer. (2018) 143:333–42. 10.1002/ijc.31326 29453769 PMC6099271

[34] Uijterwaal MH Polman NJ Van Kemenade FJ Van Den Haselkamp S Witte BI Rijkaart D. 5-year cervical (Pre) cancer risk of women screened by HPV and cytology testing. Cancer Prev Res. (2015) 8:502–8. 10.1158/1940-6207.CAPR-14-0409 25776933

[35] Thomsen LT Frederiksen K Munk C Junge J Iftner T Kjaer SK. Long-term risk of cervical intraepithelial neoplasia grade 3 or worse according to high-risk human papillomavirus genotype and semi-quantitative viral load among 33,288 women with normal cervical cytology. Int J Cancer. (2015) 137:193–203. 10.1002/ijc.29374 25471319

[36] Farnsworth A Roberts JM Garland SM Crescini J Kaldor JM Machalek DA. Detection of high-grade cervical disease among women referred directly to colposcopy after a positive HPV screening test varies with age and cytology findings. Int J Cancer. (2020) 147:3068–74. 10.1002/ijc.33128 32484236

[37] Lazare C Xiao S Meng Y Wang C Li W Wang Y. Evaluation of cervical intraepithelial neoplasia occurrence following the recorded onset of persistent high-risk human papillomavirus infection: a retrospective study on infection duration. Front Oncol. (2019) 9:976. 10.3389/fonc.2019.00976 31632909 PMC6779720

[38] Demarco M Hyun N Carter-Pokras O Raine-Bennett TR Cheung L Chen X. A study of type-specific HPV natural history and implications for contemporary cervical cancer screening programs. EClinicalMedicine. (2020) 22:293.. 10.1016/j.eclinm.2020.100293 32510043 PMC7264956

[39] Bonde J Bottari F Iacobone AD Cocuzza CE Sandri MT Bogliatto F. Human papillomavirus same genotype persistence and risk: a systematic review. J Low Genit Tract Dis. (2021) 25:27–37. 10.1097/LGT.0000000000000573 33105450 PMC7748037

[40] Schettino MT Ammaturo FP Grimaldi E Legnante A Marcello A Donnarumma G. Persistent papillomavirus type-31 and type-45 infections predict the progression to squamous intraepithelial lesion. Taiwan J Obstet Gynecol. (2014) 53:494–7. 10.1016/j.tjog.2014.06.001 25510690

[41] Mitra A MacIntyre DA Marchesi JR Lee YS Bennett PR Kyrgiou M. The vaginal microbiota, human papillomavirus infection and cervical intraepithelial neoplasia: what do we know and where are we going next? Microbiome. (2016) 4:230. 10.1186/s40168-016-0203-0 27802830 PMC5088670

[42] Kyrgiou M Moscicki AB. Vaginal microbiome and cervical cancer. Semin Cancer Biol. (2022). 10.1016/j.semcancer.2022.03.005 35276341

[43] Gillet E Meys JF Verstraelen H Bosire C De Sutter P Temmerman M. Bacterial vaginosis is associated with uterine cervical human papillomavirus infection: a meta-analysis. BMC Infect Dis. (2011) 11:10. 10.1186/1471-2334-11-10 21223574 PMC3023697

[44] Liang Y Chen M Qin L Wan B Wang H A. meta-analysis of the relationship between vaginal microecology, human papillomavirus infection and cervical intraepithelial neoplasia. Infect Agent Cancer. (2019) 14:29. 10.1186/s13027-019-0243-8 31673281 PMC6815368

[45] Brusselaers N Shrestha S van de Wijgert J Verstraelen H. Vaginal dysbiosis and the risk of human papillomavirus and cervical cancer: systematic review and meta-analysis. Am J Obstet Gynecol. (2019) 221:9–18. 10.1016/j.ajog.2018.12.011 30550767

[46] Norenhag J Du J Olovsson M Verstraelen H Engstrand L Brusselaers N. The vaginal microbiota, human papillomavirus and cervical dysplasia: a systematic review and network meta-analysis. BJOG. (2020) 127:171–80. 10.1111/1471-0528.15854 31237400

[47] Mitra A MacIntyre DA Ntritsos G Smith A Tsilidis KK Marchesi JR. The vaginal microbiota associates with the regression of untreated cervical intraepithelial neoplasia 2 lesions. Nat Commun. (2020) 11:1999. 10.1038/s41467-020-15856-y 32332850 PMC7181700

[48] Lavitola G Della Corte L De Rosa N Nappi C Bifulco G. Effects on vaginal microbiota restoration and cervical epithelialization in positive HPV patients undergoing vaginal treatment with carboxy-methyl-beta-glucan. Biomed Res Int. (2020) 2020:1–8. 10.1155/2020/5476389 32420349 PMC7201736

[49] Cheung LC Egemen D Chen X Katki HA Demarco M Wiser AL. 2019 ASCCP risk-based management consensus guidelines: methods for risk estimation, recommended management, and validation. J Low Genit Tract Dis. (2020) 24:90–101. 10.1097/LGT.0000000000000528 32243306 PMC7147416

[50] Australia CC. National Cervical Screening Program: guidelines for the management of screen-detected abnormalities, screening in specific populations and investigation of abnormal vaginal bleeding. Cancer Council Australia. (2020). Available online at: https://wiki.cancer.council.australia/Guidelines:Cervical-cancer/Screening. (accessed January 2, 2020).

[51] Kawaguchi R Matsumoto K Akira S Ishitani K Iwasaku K Ueda Y. Guidelines for office gynecology in Japan: Japan society of obstetrics and gynecology (JSOG) and Japan association of obstetricians and gynecologists (JAOG) 2017 edition. J Obstet Gynaecol Res. (2019) 45:766–86. 10.1111/jog.13831 30675969

[52] Kyrgiou M Arbyn M Bergeron C Bosch FX Dillner J Jit M. Cervical screening: ESGO-EFC position paper of the European society of gynaecologic oncology (ESGO) and the European federation of colposcopy (EFC). Br J Cancer. (2020) 123:510–7. 10.1038/s41416-020-0920-9 32507855 PMC7434873

[53] England. PH. Cervical screening: programme and colposcopy management. Public Health England. 2020. www.gov.uk/government/publications/cervical-screening-programme-and-colposcopy-management (accessed March 5, 2020).

[54] Polman NJ Snijders PJF Kenter GG Berkhof J Meijer C. HPV-based cervical screening: Rationale, expectations and future perspectives of the new Dutch screening programme. Prev Med. (2019) 119:108–17. 10.1016/j.ypmed.2018.12.021 30594536

[55] Goodman S Mody RR Coffey D Gorman BK Luna E Armylagos D. Negative Pap tests in women with high-grade cervical lesions on follow-up biopsies: Contributing factors and role of human papillomavirus genotyping. Diagn Cytopathol. (2018) 46:239–43. 10.1002/dc.23874 29230975

[56] Gilham C Sargent A Kitchener HC Peto J HPV. testing compared with routine cytology in cervical screening: long-term follow-up of ARTISTIC RCT. Health Technol Assess. (2019) 23:1–44. 10.3310/hta23280 31219027 PMC6600121

[57] Li J Wang W Yang P Chen J Dai Q Hua P. Analysis of the agreement between colposcopic impression and histopathological diagnosis of cervical biopsy in a single tertiary center of Chengdu. Arch Gynecol Obstet. (2021) 3:12. 10.1007/s00404-021-06012-y 33683424

[58] Pesola F Rebolj M Leeson S Dunk L Pickford L Gjini A. Introducing human papillomavirus (HPV) primary testing in the age of HPV vaccination: projected impact on colposcopy services in Wales. BJOG. (2021) 128:1226–35. 10.1111/1471-0528.16610 33247993 PMC8246959

[59] Wentzensen N Walker J Smith K Gold MA Zuna R Massad LS. A prospective study of risk-based colposcopy demonstrates improved detection of cervical precancers. Am J Obstet Gynecol. (2018) 218:604–8. 10.1016/j.ajog.2018.02.009 29462629 PMC8771458

[60] Wentzensen N Walker J Schiffman M Yang HP Zuna RE Dunn ST. Heterogeneity of high-grade cervical intraepithelial neoplasia related to HPV16: implications for natural history and management. Int J Cancer. (2013) 132:148–54. 10.1002/ijc.27577 22488167 PMC3409928

[61] Jeronimo J Bansil P Valdez M Kang LN Zhao FH Qiao YL. The influence of human papillomavirus genotypes on visual screening and diagnosis of cervical precancer and cancer. J Low Genit Tract Dis. (2015) 19:220–3. 10.1097/LGT.0000000000000088 25279978

[62] Zaal A Louwers JA Berkhof J Kocken M Ter Harmsel WA Graziosi GC. Agreement between colposcopic impression and histological diagnosis among human papillomavirus type 16-positive women: a clinical trial using dynamic spectral imaging colposcopy. BJOG. (2012) 119:537–44. 10.1111/j.1471-0528.2012.03280.x 22304443

[63] Macios A Didkowska J Wojciechowska U Komerska K Glinska P Kaminski MF. Risk factors of cervical cancer after a negative cytological diagnosis in Polish cervical cancer screening programme. Cancer Med. (2021) 10:3449–60. 10.1002/cam4.3857 33934537 PMC8124104

[64] Samimi SA Mody RR Goodman S Luna E Armylagos D Schwartz MR. Do infection patterns of human papillomavirus affect the cytologic detection of high-grade cervical lesions on papanicolaou tests? Arch Pathol Lab Med. (2017) 142:347–52. 10.5858/arpa.2016-0478-OA 29160722

[65] Asciutto KC Borgfeldt C Forslund O. 14-type HPV mRNA test in triage of HPV DNA-positive postmenopausal women with normal cytology. BMC Cancer. (2020) 20:1025. 10.1186/s12885-020-07498-6 33097006 PMC7583187

[66] Zhang SK Jia MM Zhao DM Wu ZN Guo Z Liu YL. Evaluation of p16/Ki-67 dual staining in the detection of cervical precancer and cancer in China. Cancer Epidemiol. (2019) 59:123–8. 10.1016/j.canep.2018.12.013 30739069

[67] Clarke MA Cheung LC Castle PE Schiffman M Tokugawa D Poitras N. 5-Year risk of cervical precancer following p16/Ki-67 dual-stain triage of HPV-positive women. JAMA Oncol. (2019) 5:181–6. 10.1001/jamaoncol.2018.4270 30325982 PMC6439556

[68] Wright TC Jr Stoler MH Ranger-Moore J Fang Q Volkir P Safaeian M. Clinical validation of p16/Ki-67 dual-stained cytology triage of HPV-positive women: results from the IMPACT trial. Int J Cancer. (2022) 150:461–71. 10.1002/ijc.33812 34536311 PMC9293341

[69] Li Y Fu Y Cheng B Xie X Wang X. A comparative study on the accuracy and efficacy between dalton and CINtec^®^ PLUS p16/Ki-67 dual stain in triaging HPV-positive women. Front Oncol. (2022) 11:815213. 10.3389/fonc.2021.815213 35141154 PMC8818758

[70] Lukic A De Vincenzo R Ciavattini A Ricci C Senatori R Ruscito I. are we facing a new colposcopic practice in the HPV vaccination era? Opportunities, challenges, and new perspectives. Vaccines. (2021) 9:81. 10.3390/vaccines9101081 34696189 PMC8538171

[71] Arbyn M Rezhake R Yuill S Canfell K. Triage of HPV-positive women in Norway using cytology, HPV16/18 genotyping and HPV persistence. Brit J Cancer. (2020) 122:1577–9. 10.1038/s41416-020-0787-9 32242099 PMC7250932

[72] Karube A Saito F Nakamura E Shitara A Ono N Konno M. Reduction in HPV 16/18 prevalence among young women following HPV vaccine introduction in a highly vaccinated district, Japan, 2008-2017. J Rural Med. (2019) 14:48–57. 10.2185/jrm.2986 31191766 PMC6545435

[73] Song F Yan P Huang X Wang C Qu X Du H. Triaging HPV-positive, cytology-negative cervical cancer screening results with extended HPV genotyping and p16(INK4a) immunostaining in China. BMC Infect Dis. (2021) 21:400. 10.1186/s12879-021-06109-4 33931022 PMC8086315

[74] Schiffman M Wentzensen N. The orderly incorporation of continuing technologic advances into cervical cancer screening. JNCI. (2021) 113:231–3. 10.1093/jnci/djaa106 32745173 PMC7936058

[75] Rebolj M Brentnall AR Mathews C Denton K Holbrook M Levine T. 16/18 genotyping in triage of persistent human papillomavirus infections with negative cytology in the English cervical screening pilot. Br J Cancer. (2019) 121:455–63. 10.1038/s41416-019-0547-x 31409912 PMC6738108

[76] Stoler MH Wright TC Jr Parvu V Yanson K Cooper CK Andrews J. Stratified risk of high-grade cervical disease using onclarity HPV extended genotyping in women, >/=25years of age, with NILM cytology. Gynecol Oncol. (2019) 153:26–33. 10.1016/j.ygyno.2018.12.024 30638767

[77] Stoler MH Wright TC Parvu V Yanson K Eckert K Kodsi S. HPV Testing with 16, 18, and 45 genotyping stratifies cancer risk for women with normal cytology. Am J Clin Pathol. (2019) 151:433–42. 10.1093/ajcp/aqy169 30649177 PMC6396747

[78] Khunamornpong S Settakorn J Sukpan K Suprasert P Srisomboon J Intaraphet S. Genotyping for human papillomavirus (HPV) 16/18/52/58 has a higher performance than HPV16/18 genotyping in triaging women with positive high-risk HPV Test in Northern Thailand. PLoS ONE. (2016) 11:e0158184. 10.1371/journal.pone.0158184 27336913 PMC4918932

[79] Wentzensen N Lahrmann B Clarke MA Kinney W Tokugawa D Poitras N. Accuracy and efficiency of deep-learning-based automation of dual stain cytology in cervical cancer screening. Jnci-J Natl Cancer I. (2021) 113:72–9. 10.1093/jnci/djaa066 32584382 PMC7781458

[80] Wentzensen N Clarke MA Bremer R. clinical evaluation of human papillomavirus screening with p16/Ki-67 dual stain triage in a large organized cervical cancer screening program. Jama Intern Med. (2019) 179:1007. 10.1001/jamainternmed.2019.0306 31081870 PMC6515572

[81] Gajsek US Dovnik A Takac I Ivanus U Jerman T Zatler SS. Diagnostic performance of p16/Ki-67 dual immunostaining at different number of positive cells in cervical smears in women referred for colposcopy. Radiol Oncol. (2021) 55:426–32. 10.2478/raon-2021-0043 34821133 PMC8647795

[82] Wright TC Behrens CM Ranger-Moore J Rehm S Sharma A Stoler MH. Triaging HPV-positive women with p16/Ki-67 dual-stained cytology: results from a sub-study nested into the ATHENA trial. Gynecol Oncol. (2017) 144:51–6. 10.1016/j.ygyno.2016.10.031 28094038

[83] Jiang MY Wu ZN Li TY Yu LL Zhang SK Zhang X. Performance of HPV Genotyping combined with p16/Ki-67 in detection of cervical pre-cancer and cancer among HPV-positive Chinese Women. Cancer Prev Res. (2020) 13:163–71. 10.1158/1940-6207.CAPR-19-0144 31871224

[84] Fu Y Li X Li Y Lu W Xie X Wang X. Head-to-head comparison of DH3 HPV test and HC2 assay for detection of high-risk HPV infection in residual cytology samples from cervical cancer screening setting: baseline and 3-year longitudinal data. Microbiol Spectr. (2022) 10:e0157021. 10.1128/spectrum.01570-21 35171029 PMC8849094

[85] Zhao X Wu Q Wang X Fu Y Zhang X Tian X. The performance of human papillomavirus DNA detection with type 16/18 genotyping by hybrid capture in primary test of cervical cancer screening: a cross-sectional study in 10,669 Chinese women. Clin Microbiol Infect. (2018) 24:1322–7. 10.1016/j.cmi.2018.02.027 29518562

[86] Bonde J Floore A Ejegod D Vink FJ Hesselink A van de Ven PM. Methylation markers FAM19A4 and miR124-2 as triage strategy for primary human papillomavirus screen positive women: a large European multicenter study. Int J Cancer. (2021) 148:396–405. 10.1002/ijc.33320 32997803 PMC7756277

[87] Vink FJ Lissenberg-Witte BI Meijer CJLM Berkhof J van Kemenade FJ Siebers AG. FAM19A4/miR124-2 methylation analysis as a triage test for HPV-positive women: cross-sectional and longitudinal data from a Dutch screening cohort. Clin Microbiol Infec. (2021) 27:18. 10.1016/j.cmi.2020.03.018 32222459

[88] Dick S Kremer WW De Strooper LMA Lissenberg-Witte BI Steenbergen RDM Meijer C. Long-term CIN3+ risk of HPV positive women after triage with FAM19A4/miR124-2 methylation analysis. Gynecol Oncol. (2019) 154:368–73. 10.1016/j.ygyno.2019.06.002 31182225

[89] Cook DA Krajden M Brentnall AR Gondara L Chan T Law JH. Evaluation of a validated methylation triage signature for human papillomavirus positive women in the HPV FOCAL cervical cancer screening trial. Int J Cancer. (2019) 144:2587–95. 10.1002/ijc.31976 30412281 PMC6492122

[90] Dick S Vink FJ Heideman DAM Lissenberg-Witte BI Meijer C Berkhof J. Risk-stratification of HPV-positive women with low-grade cytology by FAM19A4/miR124-2 methylation and HPV genotyping. Br J Cancer. (2022) 126:259–64. 10.1038/s41416-021-01614-4 34743198 PMC8770638

